# The C-reactive protein-triglyceride-glucose index predicts new-onset atrial fibrillation after ST-segment elevation myocardial infarction

**DOI:** 10.3389/fcvm.2026.1735647

**Published:** 2026-01-27

**Authors:** Bowen Qiu, Shuzhao Xia, Caixia Liu, Liming Wang, Huicong Ma, Zenglei Han, Sihua Ding, Xiuwei Wang, Deyu Yang

**Affiliations:** 1 Department of Cardiology, The Qingdao Eighth People’s Hospital, Qingdao, China; 2 Department of Internal Medicine, Pingdu Fourth People’s Hospital, Qingdao, China; 3 Department of Medical Records, Pingdu Fourth People’s Hospital, Qingdao, China; 4 Department of Emergency and Critical Care Medicine, Qingdao Hospital, University of Health and Rehabilitation Sciences (Qingdao Municipal Hospital), Qingdao, Shandong

**Keywords:** C-reactive protein-triglyceride-glucose index, new-onset atrial fibrillation, primary percutaneous coronary intervention, ST-segment elevation myocardial infarction, TyG index

## Abstract

**Background:**

New-onset atrial fibrillation (NOAF) is a common complication following primary percutaneous coronary intervention (PCI) for ST-segment elevation myocardial infarction (STEMI), with a reported incidence of 6.3%–8.0%. It represents a significant risk factor for major adverse cardiovascular events (MACE) in this patient population. The C-reactive protein–triglyceride-glucose index (CTI), a composite biomarker reflecting both inflammatory (via high-sensitivity C-reactive protein, hs-CRP) and metabolic (via the triglyceride-glucose index, TyG index) pathways, has demonstrated prognostic potential for predicting MACE and mortality. Nevertheless, its specific utility in predicting NOAF among STEMI patients undergoing primary PCI has not been investigated.

**Methods:**

This retrospective study included 696 patients (mean age 63.9 ± 12.97 years) diagnosed with acute ST-elevation myocardial infarction. Clinical data were collected to calculate the TyG index = ln[TG(mg/dL) ×  FBG(mg/dL)/2] and the CTI = 0.412 × [CRP(mg/L)] + ln[(TG(mg/dL) × FBG(mg/dL))/2]. NOAF was defined as atrial fibrillation occurring for the first time within 30 days after primary PCI. Multivariate logistic regression was used to assess the association between CTI and NOAF. Receiver operating characteristic (ROC) analysis was used to evaluate the predictive value of the CTI, the TyG index and high-sensitivity hs-CRP, with area under the curve (AUC) differences being determined via DeLong's test.

**Results:**

Of 696 initially screened participants, 62 (8.9%) developed NOAF. Multivariate analysis (stepwise forward method) confirmed CTI, Age, LVEF, and IRA-RCA as independent predictors of post-primary PCI NOAF. In ROC analysis, the CTI demonstrated superior discriminative power for NOAF with an AUC of 0.741, compared to the TyG index (AUC = 0.686) and hs-CRP (AUC = 0.664), and the Delong test confirmed that these differences were statistically significant. Combining CTI with conventional clinical indicators further improved NOAF risk stratification (AUC = 0.795).

**Conclusions:**

CTI is an independent risk factor for post-primary PCI NOAF in STEMI patients. It exhibits superior predictive value for NOAF compared to hs-CRP or TyG index alone, making it a clinically useful tool for risk stratification in this patient population.

## Introduction

ST-segment elevation myocardial infarction (STEMI) is one of the main causes of sudden cardiac death worldwide (1, 2). While timely primary percutaneous coronary intervention (PCI) effectively restores blood flow to the infarct-related vessel and significantly reduces in-hospital mortality, postoperative complications continue to pose a significant challenge to long-term patient outcomes (3–5). One such complication is New-onset atrial fibrillation (NOAF), a common and severe arrhythmia that occurs in approximately 6.3% to 8.0% of STEMI cases (6, 7). NOAF can induce rapid ventricular rates and disrupt haemodynamic stability, thereby precipitating or exacerbating heart failure (8). It also markedly increases patients' long-term mortality and risk of major adverse cardiovascular events (9). Consequently, the early identification of high-risk NOAF patients and the implementation of proactive interventions are of significant clinical importance.

In recent years, researchers have focused on identifying predictors of NOAF in patients with STEMI following primary PCI (10, 11). There is substantial evidence indicating that inflammation and metabolic dysfunction play central roles in the pathophysiology of NOAF (12–14). Elevated levels of inflammatory markers, such as high-sensitivity C-reactive protein (hs-CRP), are closely associated with the onset and maintenance of atrial fibrillation (15). This may be due to mechanisms involving atrial fibrosis and electrical remodelling, which are induced by inflammatory cell infiltration (16). Concurrently, the triglyceride-glucose index (TyG index), a reliable, non-invasive indicator of insulin resistance, has been shown to be associated with an increased risk of atrial fibrillation (14, 17, 18). It promotes the formation of atrial fibrillation substrate by influencing left atrial remodelling and autonomic nervous system activity (19, 20).

However, the predictive efficacy of individual inflammatory or metabolic markers is often limited (14, 15). It is important to note that inflammatory and metabolic pathways do not exist in isolation within the body, but rather form a complex network of interactions that collectively exacerbate atrial remodelling (20). Based on this, we hypothesise that a composite indicator capable of integrating both inflammatory and metabolic states simultaneously may offer superior predictive value compared to any single marker. The C-reactive protein-triglyceride glucose index (CTI) is an emerging composite metric comprising hs-CRP and the TyG index. Recent studies have confirmed that the CTI effectively predicts major adverse cardiovascular events following primary PCI and is associated with cardiovascular outcomes in patients with stroke or hypertension, as well as in-hospital mortality in critically ill patients (21–23).

Nevertheless, the value of CTI in predicting post-primary PCI NOAF in STEMI patients is still unclear. This study is the first to systematically investigate the association between CTI and post-primary PCI NOAF in STEMI patients, and aims to evaluate its predictive efficacy for this specific complication. This will provide a novel, more integrated and robust tool for clinical risk stratification.

## Methods

### Study population

This was a single-center retrospective study conducted at Qingdao Eighth People's Hospital. We consecutively enrolled patients who underwent primary percutaneous coronary intervention (primary PCI) for ST-segment elevation myocardial infarction (STEMI) from October 2019 to March 2025. All patients were managed in accordance with the latest clinical practice guidelines for STEMI. The primary PCI procedure was performed as follows: After standard antiplatelet pretreatment (loading doses of aspirin 300 mg and clopidogrel 300 mg or ticagrelor 180 mg), femoral or radial artery access was established. Coronary angiography was performed to identify the infarct-related artery (IRA), and lesion severity was assessed using quantitative coronary angiography. Balloon angioplasty was first performed to restore initial blood flow, followed by stenting (drug-eluting stents were preferred) to achieve optimal revascularization (defined as residual stenosis <20% and TIMI flow grade 3). Post-procedural anticoagulation with unfractionated heparin or bivalirudin was administered per protocol, and dual antiplatelet therapy (DAPT) was initiated for long-term maintenance.

Inclusion criteria were: (1) Age ≥18 years; (2) Confirmed STEMI (diagnosed by typical chest pain lasting ≥20 min, dynamic ST-segment elevation ≥0.1 mV in two or more contiguous leads, and elevated cardiac troponin levels); (3) Underwent successful primary PCI.

Exclusion criteria were: (1) Severe hepatic or renal dysfunction (estimated glomerular filtration rate [eGFR] <30 mL/min/1.73 m^2^ or Child-Pugh class C liver disease); (2) Active infection, autoimmune disease, or malignant tumor; (3) History of AF; (3) Missing key clinical/laboratory data.

**Figure 1:**
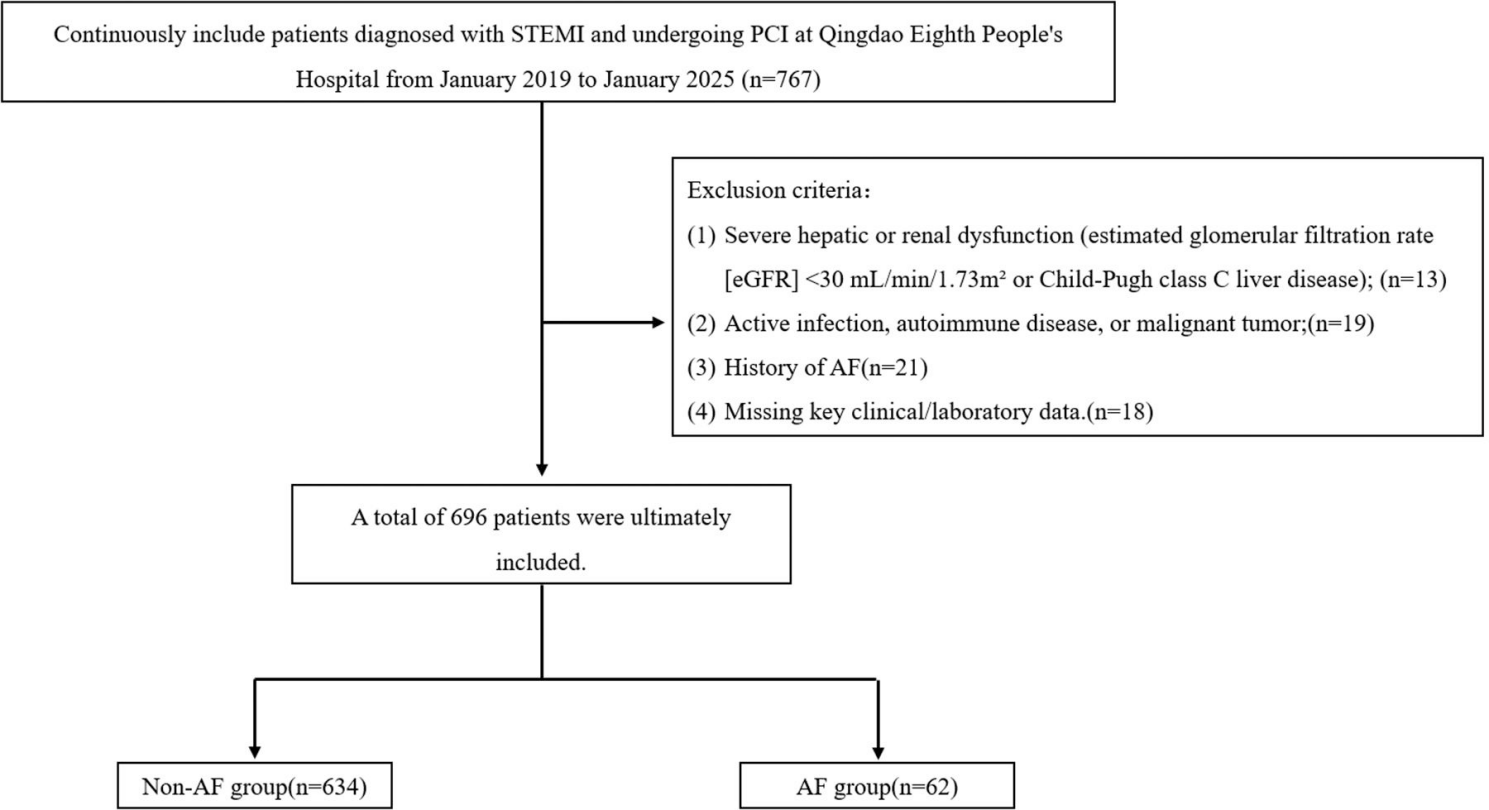
Study flowchart.

The Institutional Review Board (IRB) of Qingdao Eighth People's Hospital approved this study protocol (approval number: QBYLL-KY-2026-001). Given the low risk of the study (only routine clinical data and imaging were collected), the IRB waived the requirement for signed written consent. After applying the inclusion and exclusion criteria, 696 patients were finally included in the statistical analysis (Figure 1).

### Sample size considerations

To ensure a precise estimate of the incidence of new-onset atrial fibrillation (NOAF), a sample size calculation was performed based on the formula for estimating a population proportion. Using an anticipated incidence (p) of 8.0% (based on prior study), a margin of error (E) of ±2.5%, and a 95% confidence level (Z = 1.96), the minimum required sample size was calculated to be approximately 452. Accounting for potential incomplete data, the target sample size was set at 500.

During the study period, 696 consecutive eligible patients were enrolled, exceeding this target. The observed NOAF incidence was 8.9% (62/696), with a 95% confidence interval of 7.0% to 11.1%.

### Clinical data collection and TyG index, CTI calculation

Comprehensive clinical data were collected from electronic medical records and standardized case report forms: Demographic and baseline characteristics: Age, gender, height, weight [used to calculate body mass index (BMI) as weight/height^2^, kg/m^2^], and smoking history (current smoker, former smoker, never smoker). Past medical history: Hypertension (diagnosed by previous medical records, use of antihypertensive drugs, or blood pressure ≥140/90 mmHg on two separate occasions), diabetes mellitus [diagnosed by previous medical records, use of antidiabetic drugs/insulin, or fasting blood glucose [FBG] ≥7.0 mmol/L or glycated hemoglobin ≥6.5%], coronary heart disease (prior myocardial infarction or coronary revascularization), stroke (prior ischemic or hemorrhagic stroke confirmed by imaging), heart failure (NYHA class ≥II or left ventricular ejection fraction [LVEF] <40% before primary PCI). All diagnoses were confirmed in accordance with the corresponding international clinical guidelines. Venous blood samples were collected after an 8-hour fast on the first day of admission (before primary PCI). Tests included FBG, total cholesterol (TC), triglycerides (TG), high-density lipoprotein cholesterol (HDL-C), low-density lipoprotein cholesterol (LDL-C), white blood cell count, neutrophil count, lymphocyte count, high-sensitivity C-reactive protein (hs-CRP), platelet count, and eGFR (calculated using the CKD-EPI formula). Transthoracic echocardiography was performed within 24 h before primary PCI to measure LVEF (using the biplane Simpson's method). Oral medications prescribed before primary PCI, including antiplatelet drugs (e.g., aspirin, clopidogrel), antihypertensives (e.g., beta-blockers, ACEIs/ARBs), and lipid-lowering drugs (e.g., statins), were recorded. TyG index = ln [TG (mg/dL) × FBG (mg/dL)/2] (14, 17, 18). This index is a validated non-invasive marker of insulin resistance. =0.412 × [CRP(mg/L)] + ln[(TG(mg/dL) × FBG(mg/dL))/2] (21–23). NOAF, defined as the first occurrence of atrial fibrillation (documented by 12-lead electrocardiogram or 24 h Holter monitoring) within 30 days after primary PCI.

### Statistical analyses

Statistical analyses were performed using SPSS 27.0 (IBM Corp., Armonk, NY, USA) and R 4.3.1 (R Foundation for Statistical Computing, Vienna, Austria). A two-tailed *P* < 0.05 was considered statistically significant. Continuous variables were tested for normality using the Kolmogorov–Smirnov test. Normally distributed variables were expressed as mea*n* ± standard deviation (SD), and non-normally distributed variables as median (interquartile range, Q25–Q75). Patients were divided into two groups: NOAF group (developed NOAF post-primary PCI) and non-NOAF group (no NOAF post-primary PCI).For continuous variables: Independent samples t-test was used for normally distributed data, and Mann–Whitney U test for non-normally distributed data. For categorical variables: Chi-square (*χ*^2^) test was used; Fisher's exact test was applied if the expected frequency of any cell was <5. Univariate logistic regression analysis was performed to screen for factors associated with NOAF; variables with *P* < 0.05 in univariate analysis were included in multivariate logistic regression analysis.Multivariate logistic regression used a stepwise forward method to identify independent risk factors for NOAF, and results were expressed as odds ratios (OR) and 95% confidence intervals (CI). Receiver operating characteristic (ROC) curves were plotted for CTI, TyG index, and hs-CRP to evaluate their ability to predict NOAF. The area under the ROC curve (AUC) was compared using the DeLong test.

## Results

### Patients characteristics

A total of 696 STEMI patients undergoing successful primary PCI were analyzed, with 62 (8.9%) developing new-onset atrial fibrillation (NOAF) within 30 days post-primary PCI.

As shown in Table 1, patients with NOAF were significantly older (69.1 vs. 63.4 years, *p* < 0.001) and had higher admission heart rates (81.5 vs. 77.3 bpm, *p* = 0.028). Metabolic parameters including fasting blood glucose (6.08 vs. 5.64 mmol/L, *p* = 0.026) and TyG index (8.83 vs. 8.58, *p* < 0.001) were elevated in the NOAF group. Inflammatory and neurohormonal markers also showed significant elevations, with higher hs-CRP (5.60 vs. 3.28 mg/L, *p* < 0.001), peak NT-proBNP (3484.28 vs. 1,988.50 pg/mL, *p* = 0.001), and consequently higher CTI values (9.58 vs. 8.87, *p* < 0.001) in NOAF patients.

**Table 1:** Patient characteristics.

Variables	Total (*n* = 696)	Non-AF (*n* = 634)	AF (*n* = 62)	*P*
Age, yrs	63.90 ± 12.97	63.38 ± 13.10	69.13 ± 10.22	<.001
Sex, female	195 (28.02)	172 (27.13)	23 (37.10)	0.095
BMI, kg/m^2^	24.60 ± 3.43	24.57 ± 3.32	24.85 ± 4.43	0.635
SBP, mmHg	126.50 ± 21.72	126.61 ± 21.21	125.31 ± 26.60	0.709
DBP, mmHg	78.59 ± 13.71	78.73 ± 13.67	77.16 ± 14.19	0.39
Heart rate, bpm	77.70 ± 14.07	77.33 ± 13.88	81.45 ± 15.54	0.028
Hypertension	284 (40.80)	254 (40.06)	30 (48.39)	0.203
Diabetes mellitus	160 (22.99)	145 (22.87)	15 (24.19)	0.813
Stroke	77 (11.06)	74 (11.67)	3 (4.84)	0.102
Smoking	281 (40.37)	260 (41.01)	21 (33.87)	0.274
WBC, 10^9^/L	9.09 ± 3.27	9.03 ± 3.31	9.76 ± 2.75	0.052
N, 10^9^/L	7.89 ± 3.88	7.82 ± 3.98	8.57 ± 2.60	0.146
L, 10^9^/L	1.71 ± 1.14	1.73 ± 1.17	1.50 ± 0.66	0.133
HGB, g/L	140.50 ± 16.60	140.47 ± 16.62	140.84 ± 16.60	0.866
Plt, 10^9^/L	214.84 ± 59.15	215.81 ± 59.20	204.94 ± 58.28	0.167
Total cholesterol, mmol/L	4.41 ± 1.07	4.41 ± 1.06	4.36 ± 1.21	0.709
Triglycerides, mmol/L	1.43 ± 0.87	1.42 ± 0.90	1.51 ± 0.47	0.213
HDL-C, mmol/L	2.75 ± 0.91	2.76 ± 0.91	2.69 ± 0.97	0.567
LDL-C, mmol/L	1.06 ± 0.29	1.06 ± 0.29	1.02 ± 0.31	0.266
FBG, mmol/L	5.67 ± 1.49	5.64 ± 1.51	6.08 ± 1.29	0.026
TyG index	8.60 ± 0.61	8.58 ± 0.62	8.83 ± 0.33	<.001
hs-CRP, mg/L	3.50 (1.02, 6.80)	3.28 (0.94, 6.70)	5.60 (2.96, 10.00)	<.001
Peak hs-TnT, ng/L	3,092.50 (1,250.25, 5,637.25)	2,963.00 (1,244.25, 5,598.50)	3,516.00 (1,311.25, 5,699.00)	0.535
Peak NT-proBNP, pg/mL	2,058.02 (1,085.32, 4,294.04)	1,988.50 (1,064.82, 4,092.20)	3,484.28 (1,801.43, 5,479.40)	0.001
CTI	8.90 (8.51, 9.50)	8.87 (8.47, 9.29)	9.58 (9.25, 9.76)	<.001
LVEF	51.00 (48.75, 55.25)	51.50 (49.00, 56.00)	50.00 (41.25, 52.00)	<.001
Medication				
Aspirin	643 (92.39)	583 (91.96)	60 (96.77)	0.265
P2Y12	668 (95.98)	607 (95.74)	61 (98.39)	0.501
β-blockers	563 (80.89)	509 (80.28)	54 (87.10)	0.193
Statins	657 (94.40)	596 (94.01)	61 (98.39)	0.253
ACEI/ARB	367 (52.73)	336 (53.00)	31 (50.00)	0.652
Killip class				0.071
I	584 (83.91)	538 (84.86)	46 (74.19)	
II	46 (6.61)	41 (6.47)	5 (8.06)	
III	20 (2.87)	16 (2.52)	4 (6.45)	
IV	46 (6.61)	39 (6.15)	7 (11.29)	
IRA				
LAD	363 (52.16)	336 (53.00)	27 (43.55)	0.155
LCX	78 (11.21)	74 (11.67)	4 (6.45)	0.214
RCA	237 (34.05)	208 (32.81)	29 (46.77)	0.027

Data are presented as count (%) for categorical variables and median (Q1, Q3) or mean ± standard deviation for continuous variables.

AF, atrial fibrillation; ACEI, angiotensin-converting enzyme inhibitor; ARB, angiotensin II receptor blocker; BMI, body mass index; FBG, fasting blood glucose; HbA1c, glycated hemoglobin; HDL-C, high-density lipoprotein cholesterol; HGB, hemoglobin; HR, heart rate; hs-CRP, high-sensitivity C-reactive protein; IRA, infarct-related artery; LDL-C, low-density lipoprotein cholesterol; LVEF, left ventricular ejection fraction; PCI, percutaneous coronary intervention; PLT, platelet count; RCA, right coronary artery; TC, total cholesterol; TG, triglycerides; TyG index, triglyceride-glucose index.

Echocardiography revealed lower LVEF in the NOAF group (50.5% vs. 54.2%, *p* = 0.004), and angiography showed more frequent right coronary artery involvement as the infarct-related artery (46.8% vs. 32.8%, *p* = 0.027). No significant differences were found in other baseline characteristics including gender, BMI, comorbidities, lipid profiles, or medication use.

### Predictors of NOAF after primary PCI

In the univariate logistic regression analysis, Age, HR, FBG, Peak NT-proBNP, LVEF, Peak hs-CRP, TyG index, Killip class ≥1, IRA-RCA and CTI were found to be significantly associated with NOAF (*P* < 0.05). These variables were included in the multivariate logistic regression analysis. After using a stepwise forward method to eliminate confounding factors, the results revealed that Age, LVEF, IRA-RCA, CTI were predictors of NOAF after primary PCI (Table 2).

**Table 2:** Predictors of NOAF after primary PCI.

Variables	OR (95% CI)	*P*	OR (95% CI)	*P*
Age, yrs	1.039 (1.016–1.064)	0.001	1.044 (1.018–1.070)	<.001
Sex, female	1.584 (0.919–2.730)	0.098		
BMI, kg/m^2^	1.023 (0.949–1.103)	0.546		
Heart rate, bpm	1.021 (1.002–1.040)	0.029		
SBP, mmHg	0.997 (0.985–1.009)	0.651		
DBP, mmHg	0.992 (0.973–1.011)	0.39		
Hypertension	1.403 (0.832–2.366)	0.205		
Diabetes mellitus	1.076 (0.585–1.981)	0.813		
Stroke	0.385 (0.118–1.258)	0.114		
Smoking	0.737 (0.425–1.276)	0.276		
WBC, 10^9^/L	1.066 (0.990–1.149)	0.091		
N, 10^9^/L	1.037 (0.985–1.092)	0.167		
L, 10^9^/L	0.790 (0.583–1.072)	0.13		
HGB, g/L	1.001 (0.986–1.017)	0.866		
Plt, 10^9^/L	0.997 (0.992–1.001)	0.167		
Total cholesterol, mmol/L	0.954 (0.745–1.222)	0.709		
Triglycerides, mmol/L	1.114 (0.840–1.478)	0.452		
HDL-C, mmol/L	0.918 (0.684–1.231)	0.567		
LDL-C, mmol/L	0.536 (0.181–1.590)	0.261		
FBG, mmol/L	1.162 (1.015–1.331)	0.030		
TyG index	1.886 (1.264–2.815)	0.002		
Peak hs-CRP, mg/L	1.136 (1.069–1.206)	<.001		
Peak hs-TnT, ng/L	1.000 (1.000–1.000)	0.822		
Peak NT-proBNP, pg/mL	2.308 (1.310–4.065)	0.004		
CTI	2.261 (1.628–3.141)	<.001	2.522 (1.767–3.601)	<.001
LVEF	0.925 (0.894–0.958)	<.001	0.925 (0.891–0.960)	<.001
Aspirin	2.624 (0.624–11.044)	0.188		
P2Y12	2.713 (0.363–20.294)	0.331		
β-blockers	1.658 (0.769–3.572)	0.197		
Statins	3.889 (0.525–28.824)	0.184		
ACEI/ARB	0.887 (0.526–1.494)	0.652		
Killip class > 1	1.949 (1.060–3.584)	0.032		
IRA-LAD	0.684 (0.404–1.157)	0.157		
IRA-LCX	0.522 (0.184–1.479)	0.221		
IRA-RCA	1.800 (1.064–3.045)	0.028	1.112 (1.112–3.451)	0.02

AF, atrial fibrillation; ACEI, angiotensin-converting enzyme inhibitor; ARB, angiotensin II receptor blocker; BMI, body mass index; FBG, fasting blood glucose; HbA1c, glycated hemoglobin; HDL-C, high-density lipoprotein cholesterol; HGB, hemoglobin; HR, heart rate; hs-CRP, high-sensitivity C-reactive protein; IRA, infarct-related artery; LDL-C, low-density lipoprotein cholesterol; LVEF, left ventricular ejection fraction; PCI, percutaneous coronary intervention; PLT, platelet count; RCA, right coronary artery; TC, total cholesterol; TG, triglycerides; TyG index, triglyceride-glucose index.

### Receiver operating characteristic (ROC) analysis for identifying NOAF

**Figure 2:**
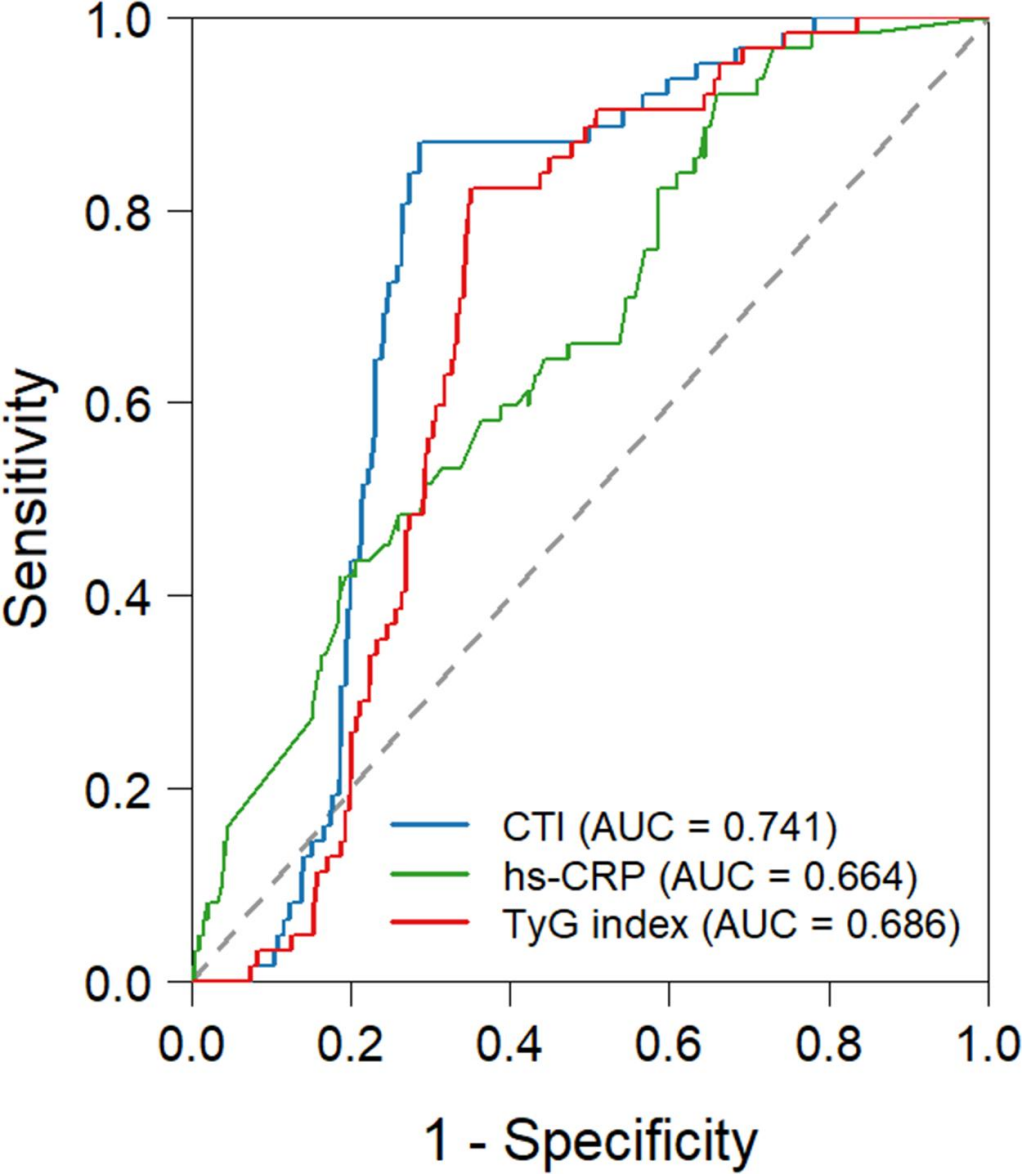
Receiver-operating characteristic curve predicting the NOAF after primary PCI. CTI, C-reactive protein-Triglyceride-glucose index; TyG index, triglyceride-glucose.

Table 3 presents the ROC analysis results of three parameters for identifying NOAF, with all *P*-values < 0.001 indicating statistical significance (Figure 2). CTI exhibited the optimal discriminative performance, with an area under the ROC curve (AUC) of 0.741 (95% CI: 0.694–0.788); at a cutoff value of 9.136, its sensitivity and specificity were 0.871 and 0.715, respectively. The TyG index showed moderate discriminative ability, with an AUC of 0.686 (95% CI: 0.638–0.735), a sensitivity of 0.823, and a specificity of 0.650 at the cutoff of 8.62. hs-CRP had the lowest discriminative efficacy, with an AUC of 0.664 (95%CI: 0.598–0.730); although it achieved a high sensitivity of 0.919 at the cutoff of 1.42, its specificity was only 0.341. The DeLong test revealed a significant difference in the area under the ROC curve (AUC) (Table 4). Furthermore, combining the CTI with conventional clinical indicators improves the stratification of NOAF risk in STEMI patients (AUC: 0.795) (Supplementary Figure S1).

**Table 3:** Receiver operating characteristic analysis of combined parameters for identifying new-onset atrial fibrillation.

Variables	AUC	95% CI	CUT-OFF	Sensitivity	Specificity	*p*
CTI	0.741	0.694–0.788	9.136	0.871	0.715	<0.001
TyG index	0.686	0.638–0.735	8.62	0.823	0.650	<0.001
hs-CRP	0.664	0.598–0.730	1.42	0.919	0.341	<0.001

CTI C-reactive protein–triglyceride glucose index; TyG, triglyceride-glucose; hs-CRP, high sensitivity C-reactive protein.

**Table 4:** Delong test.

Variables	*z*	*p*
CTI—hs-CRP	2.636	0.008
CTI—TyG index	3.043	0.002

CTI C-reactive protein–triglyceride glucose index; TyG, triglyceride-glucose; hs-CRP, high sensitivity C-reactive protein.

## Discussion

The key findings of this study are as follows: 1. The CTI is an independent predictors for NOAF in patients with STEMI following primary PCI. 2. Compared with hs-CRP and TyG index, the CTI demonstrates superior predictive power for NOAF after primary PCI in STEMI patients.

Acute myocardial infarction (AMI) has shown an increasing incidence with a younger age trend, and it is the most important cause of sudden death (3, 24). Although current treatment methods have become increasingly sophisticated, significantly reducing the in-hospital mortality of STEMI, the long-term risk of MACE in STEMI patients remains a concern (2, 25). Multiple previous studies have indicated that NOAF is an important risk factor for MACE during follow-up in STEMI patients (6, 7). Therefore, identifying relevant markers to predict NOAF after STEMI is crucial.

In recent years, composite indicators have been shown to be more stable and accurate than single markers (10, 12, 15, 26, 27). This study aims to evaluate the novel CTI index, which simultaneously encompasses systemic inflammatory status and metabolic disorder levels. Previous studies have demonstrated that the CTI is closely associated with the risk of heart failure and cardiovascular disease (CVD) (21–23). This study further reveals that the CTI can be used to effectively predict NOAF in patients with STEMI following primary PCI. Furthermore, Statistical comparisons revealed that the CTI had a significantly higher AUC than either hs-CRP alone (AUC: 0.664, 95% CI: 0.598–0.730) or the TyG index (AUC: 0.686, 95% CI: 0.638–0.735) (both *P* values < 0.05). These findings highlight the importance of the CTI in risk stratification for NOAF in patients with STEMI following primary PCI. The CTI composite index's potential superiority over traditional single markers stems from its integrated assessment of two key pathophysiological pathways: inflammation and metabolism.

Multiple existing studies have confirmed the close relationship between inflammation and cardiovascular disease (16, 28). Previous research indicates that CRP typically peaks within two days of acute myocardial infarction and correlates positively with the extent of myocardial necrosis (29–31). Sebastian J. and his colleagues demonstrated that elevated CRP levels are associated with NOAF following primary PCI in patients with STEMI (30). This relationship involves CRP binding to phosphocholine residues that are exposed on damaged cell membranes (32, 33). This activates the complement system and promotes myocardial fibrosis (33). Moreover, prior studies have demonstrated that during the acute phase of myocardial infarction, CRP accumulates at the atherosclerotic lesions within the arterial intima, thereby exacerbating microcirculatory dysfunction and intensifying myocardial ischaemia (34). These factors may significantly contribute to NOAF. Beyond CRP, other inflammatory markers and indicators of inflammatory burden also correlate with NOAF (10, 16, 27). Inflammatory cells may further promote atrial fibrillation through direct myocardial infiltration, modulation of autonomic nervous activity, and alteration of ion channel function (35, 36). Our study further demonstrates that CRP is a risk factor for NOAF following primary PCI in STEMI patients. However, as in previous studies, CRP remains a highly sensitive yet low-specificity indicator, necessitating integration with other markers to enhance its predictive value (7).

Insulin resistance promotes atrial electrical and structural remodelling by disrupting myocardial energy metabolism, impairing ion channel function and activating inflammatory pathways (37, 38). The TyG index, a commonly used non-invasive marker of insulin resistance, has been shown in several studies to be a significant risk factor for recurrence following radiofrequency ablation (17, 18). It is important to note that insulin resistance and inflammation do not exist in isolation (37). Fundamental research indicates that insulin resistance impairs the function of GLUT4 and GLUT8 transporters in cardiomyocytes, leading to the excessive production of reactive oxygen species and triggering inflammatory cascades (37). Conversely, chronic inflammation, mediated by pro-inflammatory cytokines such as TNF-α and IL-6, activates intracellular signalling pathways (e.g., the JNK and IKKβ/NF-κB pathways), thereby disrupting insulin receptor substrate phosphorylation and exacerbating insulin resistance (39, 40). This creates a vicious cycle. Compared to individual markers of inflammation or insulin resistance, the CTI index reflects both pathological states simultaneously, potentially conferring greater predictive value for NOAF.

Consistent with previous studies, we identified left ventricular ejection fraction (LVEF), Age and the IRA-RCA as independent risk factors for NOAF (11, 30). Furthermore, combining the CTI with conventional clinical indicators improves the stratification of NOAF risk in STEMI patients (AUC: 0.795) (Supplementary Figure S1).

Of course, this study has certain limitations. Firstly, as it is a single-centre retrospective study, it cannot establish causal relationships and the conclusions drawn may be affected by unmeasured confounding factors and selection bias. Secondly, CTI was measured at a single admission time point, which means that dynamic changes in inflammation and metabolism were not captured. Thirdly, monitoring for atrial fibrillation relied on routine electrocardiography, which could have resulted in asymptomatic or paroxysmal cases being overlooked. Fourthly, the absence of imaging data on atrial structure and function limited in-depth mechanistic interpretation. Finally, the incremental value and external validity of CTI relative to existing clinical prediction models have yet to be validated.

## Conclusions

CTI is an independent risk factor for post-primary PCI NOAF in STEMI patients. It exhibits superior predictive value for NOAF compared to hs-CRP or TyG index alone, making it a clinically useful tool for risk stratification in this patient population.

## Data Availability

The raw data supporting the conclusions of this article will be made available by the authors, without undue reservation.

## Supplementary material

Supplementary Figure S1 ROC curves of CTI alone and in combination with other indicators for predicting NOAF after primary PCI.

## References

[1] Zeijlon R Chamat J Enabtawi I Jha S Mohammed MM Wågerman J Risk of in-hospital life-threatening ventricular arrhythmia or death after ST-elevation myocardial infarction vs. the Takotsubo syndrome. ESC Heart Fail. (2021) 8(2):1314–23. 10.1002/ehf2.13208 33511788 PMC8006718

[2] Zoni CR D'Imperio H Zapata G Charask A Macín SM Castillo Costa Y Heart failure at admission complicating ST-elevation myocardial infarction in a middle-income country. Experience of the ARGEN-IAM-ST registry. Curr Probl Cardiol. (2024) 49(1 Pt B):102076. 10.1016/j.cpcardiol.2023.102076 37716540

[3] Zinoviev R Kumar A Huded CP Johnson M Kravitz K Reed GW Association of a comprehensive ST-segment-elevation myocardial infarction protocol with key process metrics among patients transferred for primary percutaneous coronary intervention. J Am Heart Assoc. (2025) 14(9):e034054. 10.1161/JAHA.123.034054 40314356 PMC12184277

[4] Rao SV O'Donoghue ML Ruel M Rab T Tamis-Holland JE Alexander JH 2025 ACC/AHA/ACEP/NAEMSP/SCAI guideline for the management of patients with acute coronary syndromes: a report of the American college of cardiology/American heart association joint committee on clinical practice guidelines. Circulation. (2025) 151(13):e771–862. 10.1161/CIR.0000000000001328 40014670

[5] Byrne RA Rossello X Coughlan JJ Barbato E Berry C Chieffo A 2023 ESC guidelines for the management of acute coronary syndromes. Eur Heart J. (2023) 44(38):3720–826. 10.1093/eurheartj/ehad191 37622654

[6] Jabre P Roger VL Murad MH Chamberlain AM Prokop L Adnet F Mortality associated with atrial fibrillation in patients with myocardial infarction: a systematic review and meta-analysis. Circulation. (2011) 123(15):1587–93. 10.1161/CIRCULATIONAHA.110.986661 21464054 PMC3082773

[7] Wong CK White HD Wilcox RG Criger DA Califf RM Topol EJ Significance of atrial fibrillation during acute myocardial infarction, and its current management: insights from the GUSTO-3 trial. Card Electrophysiol Rev. (2003) 7(3):201–7. 10.1023/B:CEPR.0000012382.81986.47 14739713

[8] McManus DD Hsu G Sung SH Saczynski JS Smith DH Magid DJ Atrial fibrillation and outcomes in heart failure with preserved versus reduced left ventricular ejection fraction. J Am Heart Assoc. (2013) 2(1):e005694. 10.1161/JAHA.112.005694 23525446 PMC3603249

[9] Rene AG Généreux P Ezekowitz M Kirtane AJ Xu K Mehran R Impact of atrial fibrillation in patients with ST-elevation myocardial infarction treated with percutaneous coronary intervention [from the HORIZONS-AMI (harmonizing outcomes with revascularization and stents in acute myocardial infarction) trial]. Am J Cardiol. (2014) 113(2):236–42. 10.1016/j.amjcard.2013.09.016 24176066

[10] Wang F Sun Y Lu Y Pan D An N Liu R Predictive value of platelet-to-albumin ratio combined with the C(2)HEST score for new-onset atrial fibrillation in elderly patients with acute ST-segment elevation myocardial infarction. BMC Cardiovasc Disord. (2024) 24(1):521. 10.1186/s12872-024-04200-7 39333846 PMC11429877

[11] Chen L Zhang M Chen W Li Z Wang Y Liu D Cardiac MRI left atrial strain associated with new-onset atrial fibrillation in patients with ST-segment elevation myocardial infarction. J Magn Reson Imaging. (2023) 58(1):135–44. 10.1002/jmri.28491 36326149

[12] Bao J Gao Z Hu Y Liu W Ye L Wang L. Serum fibrinogen-to-albumin ratio predicts new-onset atrial fibrillation risk during hospitalization in patients with acute myocardial infarction after percutaneous coronary intervention: a retrospective study. BMC Cardiovasc Disord. (2023) 23(1):432. 10.1186/s12872-023-03480-9 37658287 PMC10474692

[13] Pan L Li Z Li C Dong X Hidru TH Liu F Stress hyperglycemia ratio and neutrophil to lymphocyte ratio are reliable predictors of new-onset atrial fibrillation in patients with acute myocardial infarction. Front Cardiovasc Med. (2022) 9:1051078. 10.3389/fcvm.2022.1051078 36440053 PMC9681791

[14] Ling Y Fu C Fan Q Liu J Jiang L Tang S. Triglyceride-glucose index and new-onset atrial fibrillation in ST-segment elevation myocardial infarction patients after percutaneous coronary intervention. Front Cardiovasc Med. (2022) 9:838761. 10.3389/fcvm.2022.838761 35345486 PMC8957253

[15] Zahler D Merdler I Rozenfeld KL Shenberg G Milwidsky A Berliner S C-Reactive protein velocity and the risk of new onset atrial fibrillation among ST elevation myocardial infarction patients. Isr Med Assoc J. (2021) 23(3):169–73. 33734630

[16] Zeng Q Xu T Luo Z Zhou H Duan Z Xiong X Effect of inflammatory factors on myocardial infarction. BMC Cardiovasc Disord. (2024) 24(1):538. 10.1186/s12872-024-04122-4 39375629 PMC11457337

[17] Simental-Mendía LE Rodríguez-Morán M Guerrero-Romero F. The product of fasting glucose and triglycerides as surrogate for identifying insulin resistance in apparently healthy subjects. Metab Syndr Relat Disord. (2008) 6(4):299–304. 10.1089/met.2008.0034 19067533

[18] Guerrero-Romero F Simental-Mendía LE González-Ortiz M Martínez-Abundis E Ramos-Zavala MG Hernández-González SO The product of triglycerides and glucose, a simple measure of insulin sensitivity. Comparison with the euglycemic-hyperinsulinemic clamp. J Clin Endocrinol Metab. (2010) 95(7):3347–51. 10.1210/jc.2010-0288 20484475

[19] Karam BS Chavez-Moreno A Koh W Akar JG Akar FG. Oxidative stress and inflammation as central mediators of atrial fibrillation in obesity and diabetes. Cardiovasc Diabetol. (2017) 16(1):120. 10.1186/s12933-017-0604-9 28962617 PMC5622555

[20] Azarboo A Behnoush AH Vaziri Z Daneshvar MS Taghvaei A Jalali A Assessing the association between triglyceride-glucose index and atrial fibrillation: a systematic review and meta-analysis. Eur J Med Res. (2024) 29(1):118. 10.1186/s40001-024-01716-8 38347644 PMC10860290

[21] Ying Q He F Wu L Wei Q Xu J. C-reactive protein-triglyceride glucose index predicts mortality in cardiovascular-kidney-metabolic syndrome stage 0–3: a prospective cohort study. Diabetol Metab Syndr. (2025) 17(1):382. 10.1186/s13098-025-01947-7 41044646 PMC12495607

[22] Xu Y Chen S Zhu J Wang Q Li W Pan G C-reactive protein-triglyceride glucose index and stroke risk in early cardiovascular-kidney-metabolic syndrome: a national cohort study. BMC Cardiovasc Disord. (2025) 25(1):634. 10.1186/s12872-025-05143-3 40859123 PMC12382007

[23] Tang S Wang H Li K Chen Y Zheng Q Meng J C-reactive protein-triglyceride glucose index predicts stroke incidence in a hypertensive population: a national cohort study. Diabetol Metab Syndr. (2024) 16(1):277. 10.1186/s13098-024-01529-z 39574139 PMC11580337

[24] Raphael CE Sandoval Y Beachey JD Roger V Singh M Johnson MP Causes of myocardial infarction in younger patients: troponin-elevation in persons ≤65 years old in Olmsted county. J Am Coll Cardiol. (2025) 86(12):877–88. 10.1016/j.jacc.2025.07.012 40864006 PMC12445014

[25] Abdelaziz A Ramadan S Hasan MT Desouky M Atta K Hafez A Complete revascularization in patients with acute myocardial infarction and multivessel disease: pooled analysis of Kaplan-Meier-derived individual-patient-data. Am Heart J. (2026) 292:107284. 10.1016/j.ahj.2025.107284 41046115

[26] Bağcı A Aksoy F. Systemic immune-inflammation index predicts new-onset atrial fibrillation after ST elevation myocardial infarction. Biomark Med. (2021) 15(10):731–9. 10.2217/bmm-2020-0838 34155910

[27] Liu K Tao Z Li G Li M Yin J Zhou L. Predictive value of inflammatory burden index for new-onset atrial fibrillation in STEMI patients. Front Cardiovasc Med. (2025) 12:1599152. 10.3389/fcvm.2025.1599152 41035697 PMC12479414

[28] Băghină RM Crișan S Luca S Pătru O Lazăr MA Văcărescu C Association between inflammation and new-onset atrial fibrillation in acute coronary syndromes. J Clin Med. (2024) 13:5088. 39274304 10.3390/jcm13175088 PMC11396258

[29] Dong Y Wang X Zhang L Chen Z Zheng C Wang J High-sensitivity C reactive protein and risk of cardiovascular disease in China-CVD study. J Epidemiol Community Health. (2019) 73(2):188–92. 10.1136/jech-2018-211433 30530521

[30] Aronson D Boulos M Suleiman A Bidoosi S Agmon Y Kapeliovich M Relation of C-reactive protein and new-onset atrial fibrillation in patients with acute myocardial infarction. Am J Cardiol. (2007) 100(5):753–7. 10.1016/j.amjcard.2007.04.014 17719315

[31] Anzai T Yoshikawa T Shiraki H Asakura Y Akaishi M Mitamura H C-reactive protein as a predictor of infarct expansion and cardiac rupture after a first Q-wave acute myocardial infarction. Circulation. (1997) 96(3):778–84. 10.1161/01.CIR.96.3.778 9264482

[32] Kitsis RN Jialal I. Limiting myocardial damage during acute myocardial infarction by inhibiting C-reactive protein. N Engl J Med. (2006) 355(5):513–5. 10.1056/NEJMcibr063197 16885557

[33] Lagrand WK Visser CA Hermens WT Niessen HWM Verheugt FWA Wolbink G-J C-reactive protein as a cardiovascular risk factor: more than an epiphenomenon? Circulation. (1999) 100(1):96–102. 10.1161/01.CIR.100.1.96 10393687

[34] Tong DC Whitbourn R MacIsaac A Wilson A Burns A Palmer S High-sensitivity C-reactive protein is a predictor of coronary microvascular dysfunction in patients with ischemic heart disease. Front Cardiovasc Med. (2018) 4:81. 10.3389/fcvm.2017.00081 29376057 PMC5770395

[35] Hu YF Chen YJ Lin YJ Chen S-A. Inflammation and the pathogenesis of atrial fibrillation. Nat Rev Cardiol. (2015) 12(4):230–43. 10.1038/nrcardio.2015.2 25622848

[36] Ihara K Sasano T. Role of inflammation in the pathogenesis of atrial fibrillation. Front Physiol. (2022) 13:862164. 10.3389/fphys.2022.862164 35492601 PMC9047861

[37] Luo M Wang Y. The triglyceride-glucose index: a clinical tool to quantify insulin resistance as a metabolic myocardial remodeling bridge in atrial fibrillation. Biomedicines. (2025) 13(10):2348. 10.3390/biomedicines13102348 41153635 PMC12561394

[38] Qin S Luo Y Hou J Tian J Tang Y Feng Q Insulin resistance, left atrial anatomical remodeling, and recurrence in patients with atrial fibrillation undergoing radiofrequency ablation. Eur J Med Res. (2025) 30(1):819. 10.1186/s40001-025-03093-2 40877999 PMC12395672

[39] Xiao X Zhao J Ye G Li H Huang X. Free fatty acids and LPS synergistically promote macrophage M1 polarization and insulin resistance via FTO-mediated CSF1 degradation. Inflammation. (2026). 10.1007/s10753-025-02369-3 41504803 PMC12847160

[40] Lonardo A Weiskirchen R. Insulin resistance at the crossroads of metabolic inflammation, cardiovascular disease, organ failure and cancer. Biomolecules. (2025) 15(12):1745. 10.3390/biom15121745 41463398 PMC12730331

