# Supplementary figures and images for: The C-reactive protein-triglyceride-glucose index predicts new-onset atrial fibrillation after ST-segment elevation myocardial infarction
Source: Front Cardiovasc Med. 2026 Jan 27;13:1735647. doi: 10.3389/fcvm.2026.1735647 (PMC12887698; doi:10.3389/fcvm.2026.1735647)

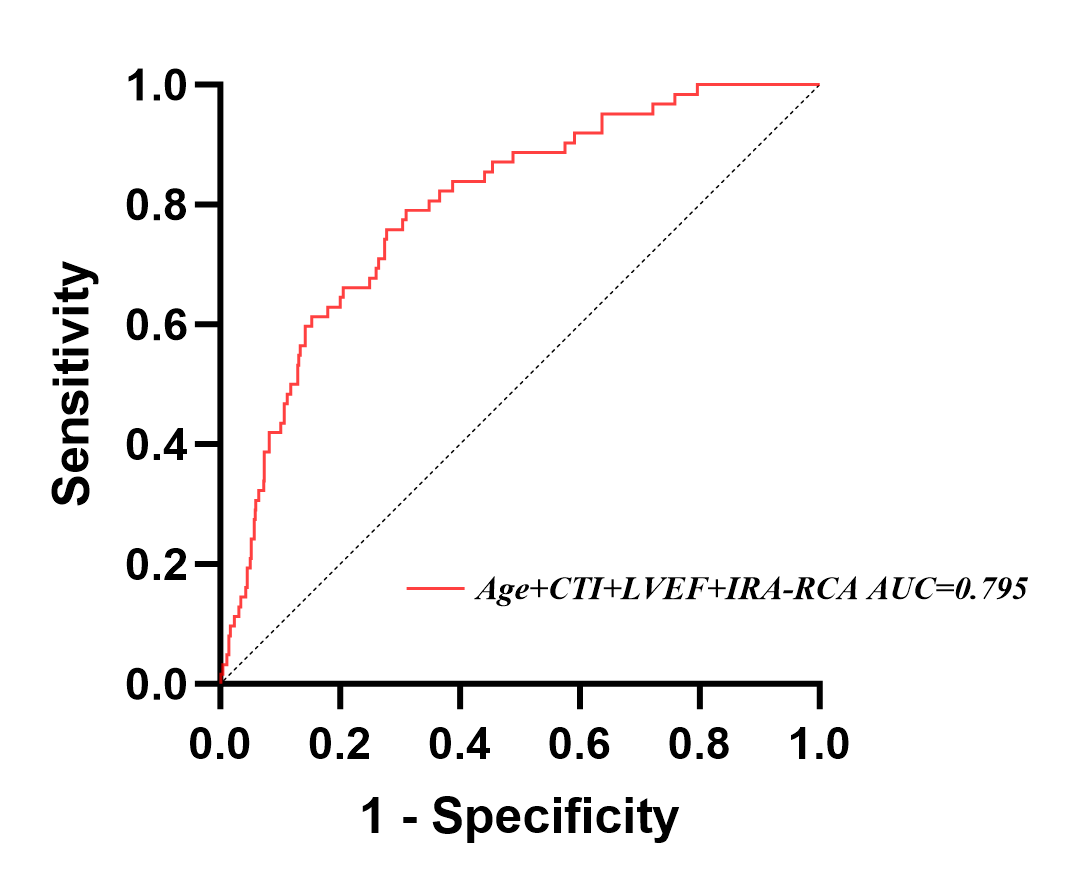

Supplement: Supplementary Figure S1 — ROC curves of CTI alone and in combination with other indicators for predicting NOAF after primary PCI. [file Image1.tif]
